# Identification and characterization of neutrophil heterogeneity in sepsis

**DOI:** 10.1186/s13054-021-03481-0

**Published:** 2021-02-06

**Authors:** Xinxin Qi, Yao Yu, Ran Sun, Jiamin Huang, Lu Liu, Yunxi Yang, Tao Rui, Bingwei Sun

**Affiliations:** 1 School of Medicine, Jiangsu University, Zhenjiang, 212001 Jiangsu Province China; 2 Department of Burns and Plastic Surgery, Affiliated Suzhou Hospital of Nanjing Medical University, Suzhou, 215002 Jiangsu Province China; 3 Center for Critical Illness Research, Lawson Health Research Institute, 800 Commisioners Road East, London, ON N6A 4G5 Canada

**Keywords:** Inhibitory neutrophil, Dysfunction, PD-L1, Immunosuppression, Sepsis

## Abstract

**Background:**

Although the immune function of neutrophils in sepsis has been well described, the heterogeneity of neutrophils remains unclear during the process of sepsis.

**Methods:**

In this study, we used a mouse CLP model to simulate the clinical scenario of patients with sepsis, neutrophil infiltration, abnormal distribution and dysfunction was analyzed. LPS was used to stimulate neutrophils in vitro to simulate sepsis; single-cell gene sequencing technology was used to explore the immunological typing. To explore the immunological function of immunosuppressive neutrophils, PD-L1 knockout neutrophils were cocultured with lymphocytes from wild-type mice.

**Results:**

We found that neutrophils presented variant dysfunction at the late stage of sepsis, including inhibition of apoptosis, seriously damaged chemotaxis and extensive infiltration into the tissues. Single-cell RNA sequencing revealed that multiple subclusters of neutrophils were differentiated after LPS stimulation. The two-dimensional spatial distribution analysis showed that Foxp3^+^ T cells were much closer to Ly-6G than the CD4^+^ and CD8^+^ cells, indicating that infiltrated neutrophils may play immunomodulatory effect on surrounding T-regs. Further observations showed that LPS mediates PD-L1 over expression through p38α-MSK1/-MK2 pathway in neutrophils. The subsets of highly expressed PD-L1 exert immunosuppressive effect under direct contact mode, including inhibition of T cell activation and induction of T cell apoptosis and trans-differentiation.

**Conclusions:**

Taken together, our data identify a previously unknown immunosuppressive subset of neutrophils as inhibitory neutrophil in order to more accurately describe the phenotype and characteristics of these cells in sepsis.

## Introduction

Sepsis is a condition with high mortality and morbidity rates and is severe in bacterial infections, epidemic virus infections (such as severe COVID-19 [1] infection), especially in intensive care patients [2]. Many factors, such as genetics, age, sex, ethnicity, the number of dysfunctional organs, immune status and therapeutic efficacy, have been linked to survival outcomes in severe sepsis. With further research, the pathophysiology of sepsis has been gradually unveiled. However, the lack of effective clinical treatments leads to high mortality [3–5].

Immunosuppression may occur for a number of reasons related to an individual’s frailty, disease or an inadequate response to infectious agents and a markedly increased prevalence of acute inflammatory-, sepsis- and infection-related death [6, 7]. Therefore, the balance of the immune system is essential for bacterial clearance and timely resolution of sepsis. At the early stage of infection, the immune system is activated and can effectively defend against various pathogenic factors, including microorganisms. However, dysfunction of the immune system gradually appears with the progression of the disease and causes immunosuppression [6, 8]. Immunosuppression is rather complicated and occurs in both cellular and humoral immunity. At present, understanding of the innate immune system in the occurrence and development of sepsis is further deepening, but many questions regarding the pathological mechanism remain [9].

Neutrophils play a critical role in eliminating invading bacteria and are considered the main defense at the early stage of infection [10]. Extensive studies both in basic and clinical research have identified that neutrophil dysfunction damages the innate immune system [7, 11]. Dysregulated immune responses to infection and subsequent tissue injury underlie sepsis pathobiology [3]. In practice, sepsis patients are only monitored by clinical criteria and peripheral blood leukocyte counts without detailed assessment of the immune system, especially the innate immune system. Notably, as neutrophils are capable of phagocytizing bacteria, the reason why a significant and persistent increase in neutrophils means that the disease is serious is worth considering. We believe that the key mechanisms of neutrophil dysfunction during sepsis remain unclear. Further in-depth assessment of neutrophil activation and immunological function may enable new therapeutic strategies for the management of human sepsis and inflammatory diseases.

Our previous research showed that neutrophil chemotaxis is seriously damaged in sepsis [12]. We found that LPS was a potent stop signal for chemotactic neutrophil migration. Treatment with an antagonist of the ATP receptor (P2X1) in primary human neutrophils or knockout of the P2X1 receptor in neutrophil-like differentiated HL-60 (dHL-60) cells recovered neutrophil chemotaxis. Additionally, LPS-induced ATP release through connexin 43 (Cx43) hemichannels activated the P2X1 receptor and the subsequent calcium influx. Increased intracellular calcium inhibited neutrophil chemotaxis by activating myosin light chain (MLC) through the myosin light chain kinase (MLCK)-dependent pathway. Damaged chemotaxis causes neutrophil infiltration into all organs, leading to organ dysfunction and even multiple organ dysfunction syndrome (MODS) [13].

However, what is the outcome of extensive neutrophil infiltration in organs and tissues? How do infiltrating neutrophils modulate the immune system? We conducted a thorough study on this topic. In this work, we found that in the late stages of sepsis, neutrophils exhibit significant heterogeneity, dysfunction and immunosuppressive effects, which inhibit T cell functions and induce T cell transdifferentiation through the PD-L1 pathway. Therefore, we defined a previously unknown immunosuppressive subset with PD-L1 expression as inhibitory neutrophils to more accurately describe the phenotype and characteristics of these cells and contribute to the study of immune system abnormalities in sepsis.

## Materials and methods

### Ethical statement

This study was approved by The Medical Ethical Committee of Nanjing Medical University. For experiments involving human blood samples, signed informed consent was obtained from all healthy volunteers. Blood samples were taken from the cubital veins of healthy donors. All the experimental methods were carried out in accordance with the approved guidelines. All experimental procedures involving mice were carried out in strict accordance with the recommendations in the Guide for the Care and Use of Laboratory Animals of the National Institutes of Health and State Key Laboratory of Pathogens and Biosecurity of the Institute of Microbiology and Epidemiology.

### Animals

In total, 8–10-week-old male C57BL/6 were purchased from Jiangsu University. PD-L1^−/−^ mice (8–10 weeks old, C57BL/6) mice were purchased from the Beijing Biocytogen Co. Ltd. Mice were raised under specific sterile conditions at the Jiangsu University School of Medicine Animal Resource Center under standard conditions at unaltered room temperature, humidity and a regular light/dark cycle. Animals were raised a standard diet and had open access to tap water. The feeding practices were approved by the animal care and use committee (IACUC) of the Jiangsu University School of Medicine. In all experiments, animals were euthanized after surgery.

### Human neutrophils chemotaxis assay

The isolation and chemotaxis assay of neutrophils were performed in the manner previously reported [14]. Ficoll–Paque (GE Healthcare, USA) density centrifugation was used to extract neutrophils from the peripheral blood of healthy volunteers. Neutrophils were placed into the side wells of the agarose chemotaxis model, and the middle well was filled with fMLP. Chemotaxis distances were observed and labeled with a microscope.

### Isolation of neutrophils from bone marrow

Neutrophils were isolated as previously described [15]. Briefly, the femur and the tibia were removed and freed of soft tissue attachments. HBSS–EDTA solution was forced through the bone with a syringe. After centrifugation, cells were then placed in a three-layer Percoll gradient of 78%, 69% and 52% (GE Healthcare, USA) and collected the cells from the 78%/69% interface. We obtained 6 ± 1 × 10^6^ cells per mouse and purity greater than 95% (Ly-6G was used as the surface marker).

### Neutrophil phagocytosis assay

Neutrophil phagocytic activity was detected using flow cytometry (FCM). Neutrophils with or without LPS were incubated with *E. coli* ATCC 25922-GFP for 0.5 h, and the fluorescence intensity represents phagocytic activity.

### Neutrophils apoptosis

Neutrophils were washed and suspended in ice-cold PBS with 1% FBS at 2 × 10^6^ cells/mL (100 μl PBS, 2 × 10^5^ cells/tube). Antibodies and apoptosis kit (BD Bioscience) were added according to the manufacturer’s protocol. After incubating for 30 min at 4 °C in the dark, cells were washed and detected in a FACS Canto II cytometer (BD Biosciences). The data were analyzed by the FlowJo software (Tree star).

### Single-cell RNA sequencing

Neutrophils with or without LPS were cultured in vitro for 4 h and were subjected to single-cell RNA sequencing analysis (10× Genomics). The cell pellet was resuspended by 1 ml 1× PBS containing 0.04% BSA, and the washing procedure was repeated twice. After washing, obtain single-cell dispersion suspension with a concentration close to 1000 cell/μl. Then immediately place the samples on ice. During the library construction, Gel Bead and cells were wrapped by "oil droplets" to form Gel Bead in Emulsion. Cells dissolve in this reaction system and complete reverse transcription to form a full-length cDNA sequence. After the library construction, Qubit® 3.0 Fluorometer (Life Technologies, CA, USA) was used to determine the library concentration. Agilent 2100 was used to detect the fragment length distribution of the library. Also, Q-PCR technique was used to accurately quantify the effective concentration of the library. The effective concentration of the library aimed > 10 nmol/l. The qualified library was sequenced on an Illumina HiSeq platform.

### Single-cell RNA sequencing analysis, data analysis and visualization

The gene-barcode matrix obtained from Cell Ranger was analyzed using the Seurat R package version 3.1.0. Raw data were imported into the Seurat and filtered applying this threshold: cells with less than 200 or more than 3000 detected genes per cell were filtered out. Moreover, more than 20% UMIs mapped to mitochondrial genes were filtered out as well. A validation consistency procedure of the resolution allowed us to select 0.1 as the suitable resolution level. The total 6 identified clusters were visualized using UMAP as implemented in Seurat.

### Single-cell trajectory (pseudotime) analysis

Subcluster 2 and 4 cell populations were ordered in pseudotime using the Monocle 2 package. The trajectory was designed using the plot_cell_trajectory command [16]. Cells were arranged along an artificial trajectory based on gene expression changes. The trend of PD-L1 and MHC-II gene expression was depicted by individual graphs using the plot_genes_in_pseudotime function.

### Isolation of lymphocytes

Single-cell suspensions were prepared from spleens of C57BL/6 mice. Spleen tissues were pulverized and filtered through a 70-mm cell strainer (FALCON). RBCs were lysed using lysis buffer (BD Bioscience, USA). CD3 + lymphocytes were purified (> 98% purity) by negative selection with the CD3 + T cell isolation kit (BD Bioscience, USA), according to the manufacturers’ instructions.

### Lymphocyte culture and treatments

To determine the interactions of lymphocyte–neutrophil, lymphocytes (1.0 × 10^5^ per well) were plated in 96-well plates precoated with CD3 (2 μg/ml, BD Bioscience, USA) antibodies and supplemented with CD28 antibodies (5 μg/ml, BD Biosciences, USA) to reverse the hyporesponsiveness of lymphocytes. Neutrophils (2.0 × 10^5^ per well) from wild-type (WT) and PD-L1-deficient (PD-L1^−/−^) mice were added to cultures together. Transwell experiments, in which cell–cell contact between neutrophils and lymphocytes, were prevented. Neutrophils were added to the upper chambers of 24-well transwell trays, and lymphocytes were added to the lower chambers. Antibodies (BD Bioscience, USA) were added according to the manufacturer’s protocol. Changes in lymphocyte function after in vitro culture were assessed by FCM.

### Cecal ligation and puncture (CLP)

Mice were acclimated for 1 week before experiments. In order to induce polymicrobial sepsis, WT and PD-L1^−/−^ mice underwent cecal ligation and puncture (CLP) [17]. After disinfection of the abdominal wall using povidone iodine, a small midline abdominal incision was made and the cecum was exteriorized and then used silk thread to ligate under the ileocecal valve. The cecum was then punctured twice with a 27- or 23-gauge needle and gently squeezed to extrude a small amount of stool. The cecum was placed back into the peritoneal cavity, and the abdominal wall was closed in layers. Sham model mice were treated identically, but their cecums were neither punctured nor ligated. After surgery, 1 ml of 0.9% saline was injected subcutaneously. All mice were free access to food and water. Some mice were used for Kaplan–Meier survival analysis, others were killed at indicated time points after surgery, and tissue samples were taken for analysis.

### Quantitative real-time PCR

All of inhibitors (P38i, P38(αβ)i, MSK1i, MK2i) were obtained from ApexBio (USA). Total RNA was extracted using QIAzol Lysis Reagent (QIAGEN, USA), followed by a reverse transcription using the reverse transcription reagent kit (Thermo Fisher, USA). Real-time qPCR was performed using the QuantiNovaTM SYBR Green PCR kit (QIAGEN, USA) as a template to detect the mRNA expression level of each primer set. Primer Premier 5.0 software (Premier Biosoft International, USA) was used to design the primer. GAPDH was chosen as the internal reference for expression and copy number variation detection, and its expression was evaluated by the following primers: forward 5′-CACCCCATTTGATGTTAGTG-3′ and reverse 5′-CCATTTGCAGTGGCAAAG-3′. Relative expression levels were calculated using 2–DDCt method. All tests were performed in three biological repeats. The following sequences of primers are as follows: CD274-forward 5′-ACGGGCGTTTACTATCACGG-3′ and reverse 5′-CCCAGTACACCACTAACGCA-3′.

### Western Blot

All of inhibitors (P38i, P38(αβ)i, MSK1i, MK2i) were obtained from ApexBio (USA). After LPS stimulation, neutrophils were lysed in ice-cold Cell Lytic cell lysis reagent (Sigma, USA) supplemented with Phosphatase Inhibitor Cocktail 2 (Calbiochem, USA) and EDTA-free protease inhibitor cocktail (Calbiochem, USA) for 5–10 min on ice. Cell lysates were scraped from 6-well plates, collected and centrifuged for 10 min at 8000*g*. Lysates were mixed 4:1 with 5× SDS-PAGE sample loading buffer and boiled for 8 min. Samples were subjected to SDS-PAGE on 8–12% gradient gels and transferred to Amersham Hybond-ECL (GE Healthcare, USA). Membranes were blocked for 1 h in 5% Milk in PBS-Tween (w/v), then incubated in 5% BSA in PBS-Tween (w/v) with antibodies against P38 (1:1000), P38α (1:1000), MSK1 (1:1000), MK2 (1:1000), phosphorylated p38 (1:1000), phosphorylated p38α (1:1000), phosphorylated MSK1 (1: 1000), phosphorylated MK2 (1:1000) (CST, USA), PD-L1 (1:1000) (Abcam, UK) and β-actin (1:1000) (Santa Cruz Biotechnology, USA), respectively. Then the membranes were incubated with secondary antibody conjugated to HRP (1: 2000–1:5000) (CST, USA). Protein bands were developed and detected using the Pierce ECL Western Blotting Substrate (Thermo Fisher, USA).

### Immunohistochemical and immunofluorescence examination

PFA-fixed, paraffin-embedded mouse lung tissues were analyzed for each condition. In total, 5-um sections were cut and mounted on slides. Slides were deparaffinized in xylene and rehydrated in a graded series of ethanol. Antigen retrieval process was performed in 10 mM citrate buffer, in a pressure cooker for 2 min. Then endogenous peroxidases were blocked with 3% hydrogen peroxide for 20 min. Tissues were blocked in normal serum for 3 h. Then the tissues were incubated with primary antibody and followed by secondary antibody conjugated to chromogenic substrate or fluorescence. After color development and sealing, the tissues were observed with microscope. Immunohistochemical images were collected on Olympus IX71 microscope equipped with a Olympus DP 73 digital camera. Immunofluorescence images were collected on a confocal laser scanning microscope (Nikon Eclipse C1) equipped with a Nikon DS-U3 digital camera.

### Multiplexed immunofluorescence

Multiplexed immunofluorescence (IF) was performed by staining 4-um-thick formalin-fixed, paraffin-embedded whole tissue sections with standard, primary antibodies sequentially and paired with a unique fluorochrome followed by staining with DAPI. Slides were washed in Tris buffer (5 min) and then transferred to preheated citrate solution (90 °C) before being heat-treated using a microwave set at 20% of maximum power for 15 min. Slides were cooled in the same solution to room temperature. Between all steps, the slides were washed with Tris buffer. The same process was repeated for the following antibodies: anti-CD4, anti-CD8 and anti-foxP3 (CST, USA), anti-Ly6G (Abcam, UK). Add 100 µl DAPI (5 μg/ml, Sigma, USA) to each slide, washed in distilled water and manually cover slipped. Slides were air-dried, mounted with Prolong Diamond Anti-fade mounting medium and take pictures with Mantra tissue imaging system (PerkinElmer). Images were analyzed using Indica Halo software [18]**.**

### Statistical analyses

GraphPad Prism 5 was applied for the statistical analysis of all data. Values are presented as the mean ± SD. One-way factorial analysis of variance (ANOVA), Tukey’s test and Student’s t test for the comparisons were performed. A value of P < 0.05 level was considered to be significant. Significant differences were noted by asterisks (* p < 0.05, ** p < 0.01, *** p < 0.001, **** p < 0.0001).

## Results

### Neutrophil infiltration, abnormal distribution and dysfunction are associated with the progression of sepsis

**Fig. 1:**
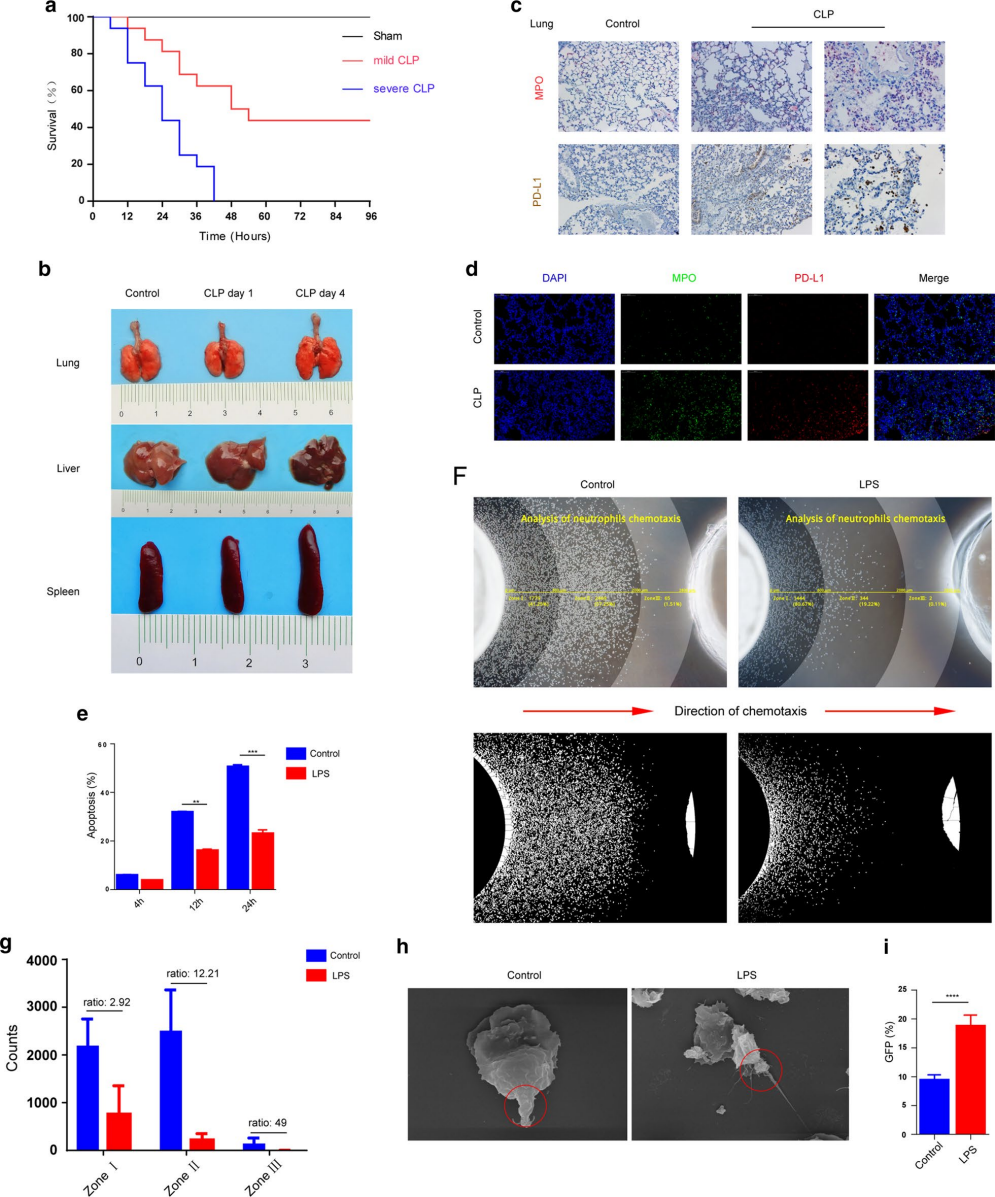
Sepsis results in neutrophil function challenge and increasing in neutrophil expression of PD-L1. **a** Survival rate change in CLP mouse model of sepsis. C57BL6/J mice with CLP-induced sepsis were randomized in three groups: sham (n = 12), severe CLP group (n = 16) and mild CLP group (n = 16). **b** Lung, liver and spleen pictographs from sham animals (control) and animals challenged with cecal-ligation and puncture (CLP). **c**, **d** Immunohistochemistry and immunofluorescence changes in lung sections of CLP (96 h) sepsis mouse model. In the immunohistochemistry, MPO was marked in red and PD-L1 expressing was marked in brown. In immunofluorescence, DAPI showed the cell nucleus in light blue, MPO marked the neutrophils in green, and PD-L1 expressing was marked in red. **e** Changes in mouse neutrophil apoptosis result after LPS stimulation (n = 5). **f**–**g** Neutrophil chemotaxis toward fMLP was assayed (n = 6). **h** Scanning electron microscopy showed shape change of neutrophils after LPS stimulation. The red circles represent the posterior tails of polarized neutrophils. **i** Changes in neutrophil phagocytosis in mice after LPS stimulation (n = 5). **p < 0.01, ***p < 0.001, ****p < 0.0001. Data are mean ± SEM

To investigate the relationship between neutrophil infiltration and the mortality rate of mice with sepsis, we used a mouse CLP model to simulate the clinical scenario of patients with sepsis. As shown in Fig. 1a, the mouse survival rate was different among the groups. In the sham procedure group, all mice were alive at 72 h after surgery. However, the mice began to die at 6 h and 12 h after surgery in the severe CLP group and mild CLP group, respectively. At 36 h, the mortality rates were 81.25% and 37.5% in mice in the severe CLP group and mild CLP group, respectively. All mice in the severe CLP group died 72 h after surgery.

Compared with mice in the sham operation group, congestion and edema appeared in lung, liver and spleen 24 h after CLP, which were more obvious at 96 h after operation (Fig. 1b). Immunohistochemical and immunofluorescence assays demonstrated significantly greater neutrophil influx into the lung organ in animals challenged with CLP 4 days group when compared to sham controls. Meanwhile, lung tissue structure of CLP group was significantly damaged, alveolar reduction, pulmonary interstitial edema. Interestingly, the expression of PD-L1 in neutrophils was significantly increased in septic mice compared with that in control mice (Fig. 1c, d, Additional file 1: Fig. S6).

To determine whether sepsis changes the biological functions of neutrophils and the underlying mechanism, we employed in vitro sepsis model. Neutrophils derived from mouse bone marrow were incubated in RPMI 1640 supplemented with 1% heat-inactivated FBS with or without LPS. Neutrophil survival was detected by flow cytometry (Fig. 1e) up to 24 h after incubation. We found that LPS challenge prolonged the survival of neutrophils compared with that of the control group. However, LPS-stimulated neutrophils showed decreased migration and reduced chemotaxis in response to fMLP (Fig. 1f). In order to accurately evaluate this phenomenon, we first divided the interwell region in the agarose neutrophil chemotaxis model into three parts (Zone I, Zone II, Zone III). Zone I represents the range of cells incubated for 1 h and is used to evaluate the initiation of neutrophils movement. Zone II is the site of most cells after incubation for 2 h and represents the overall chemotaxis of neutrophils. According to the study, in Zone I, the ratio of neutrophils in the LPS-stimulated group and the control group was 2.92, while that in Zone II and Zone III increased to 12.21 and 49, respectively (Fig. 1f, g). This is direct evidence that both initiation and persistence of neutrophil chemotaxis are decreased after LPS stimulation.

We observed the morphological characteristics of neutrophil migration with SCM. As shown in Fig. 1h, LPS administration dramatically elongated the posterior tail of the polarized neutrophil, which is essential for chemotaxis. In addition, the phagocytic ability of LPS-treated neutrophils was also studied. As shown in Fig. 1i, LPS increased neutrophil phagocytosis. The karyotype change in neutrophils is shown in Additional file 1: Fig. S1, and the number of segmented nuclei in neutrophils increased after LPS challenge. Notably, LPS increased neutrophil expression of CD274 (PD-L1), which is consistent with other stimulants of sepsis, such as IL-1β, HMGβ1, G-CSF and IL-4 (see Additional file 1: Fig. S2).

### Sepsis elicits analogous subsets of inhibitory neutrophils

**Fig. 2:**
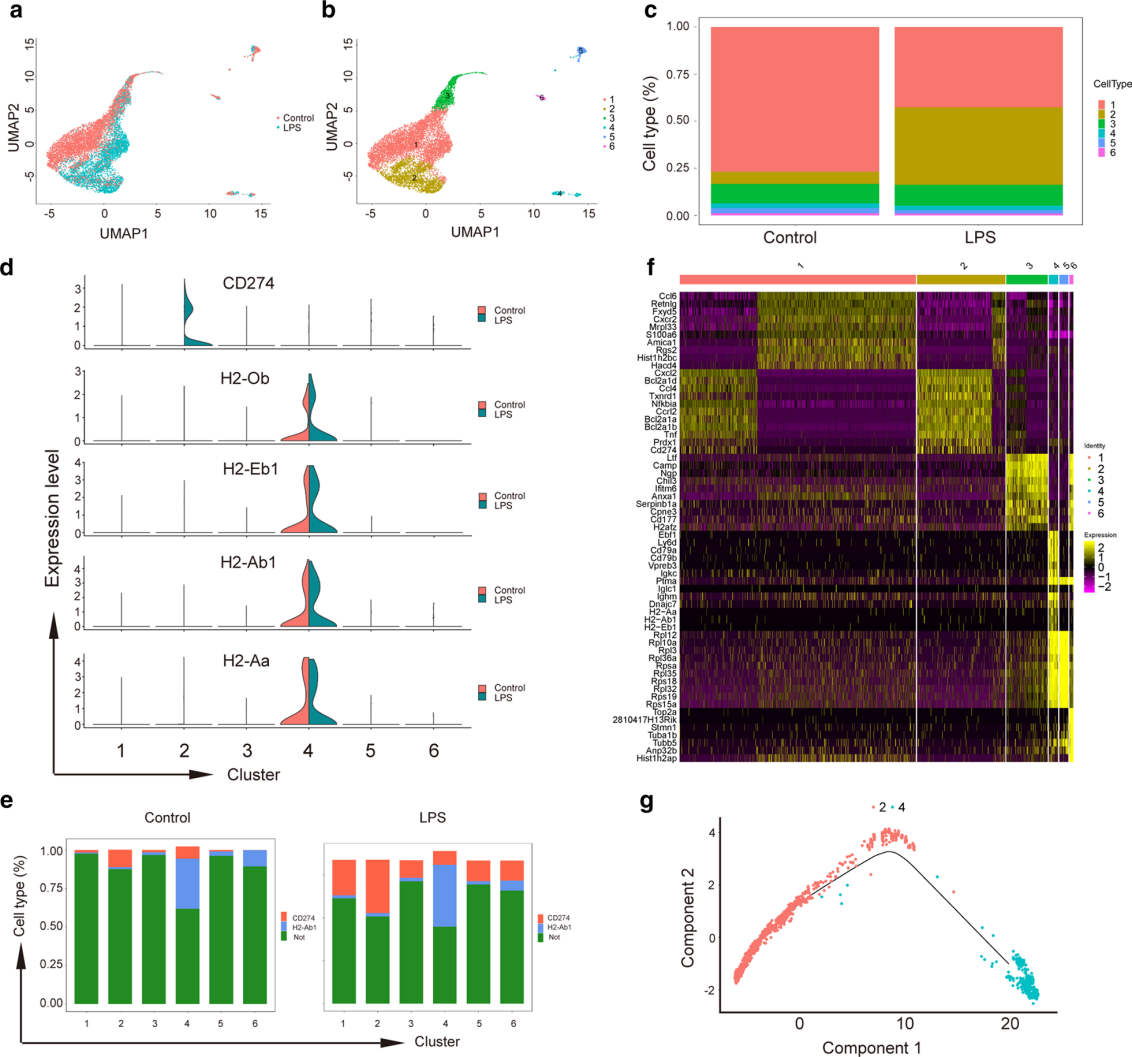
scRNA-seq uncovers neutrophil transcriptional heterogeneity after LPS stimulation. **a** UMAP representation of gene expression data in 9421 neutrophils, including the control and LPS-stimulated cells. **b** Seurat cluster assignment projected onto the UMAP plot. **c** The proportion of each subset of cells in the control group and the LPS stimulation group. **d** PD-L1 and MHC-II components (H2-Ob, H2-Eb, H2-Ab1, H2-Aa) expression in different clusters. **e** Bar plot showing the changes in the proportions of neutrophils expressing PD-L1 or MHC-II components (H2-Ab1) in each cluster. **f** Heat map of significant differential gene in each cluster. **g** Pseudotime map showed changes in neutrophil differentiation after LPS stimulation

To identify the underlying mechanism of sepsis-induced neutrophil dysfunction, we challenged neutrophils with LPS for 4 h, at which point the cells had the highest PD-L1 mRNA expression with 95% cell activity (Fig. 1e and Additional file 1: Fig. S3). LPS-challenged neutrophils were used for single-cell RNA sequencing (scRNA-seq). The profiles of neutrophils from mouse bone marrow that were challenged with LPS were different from those of the control group (Fig. 2a). We compared gene expression patterns across all neutrophils before and after LPS stimulation and identified 6 transcriptionally distinct cell clusters (Fig. 2b, c). Unsupervised clustering analysis identified major populations of cells, each of which expressed genes encoding molecules, including PD-L1 and MHC-II (Fig. 2d–f, Additional file 1: Fig. S4C-D).

PD-L1 was highly expressed in cluster 2, while MHC-II was mainly expressed in cluster 4. To further investigate the inhibitory function of neutrophils, we chose PD-L1 and MHC-II, which are hallmarks of immunoactivation and immunosuppression, as the gene biomarkers of neutrophil subtypes for the next study. By pseudotime analysis, we found that these two subtypes of neutrophils are in different trajectory differentiation stages (Fig. 2g). PD-L1 gene and MHC-II component expression were plotted as a function of pseudotime and are also shown (Additional file 1: Fig. S4B). Thus, we believe that sepsis elicits two subsets of neutrophils that can either enhance or downregulate the immune system.

**Fig. 3:**
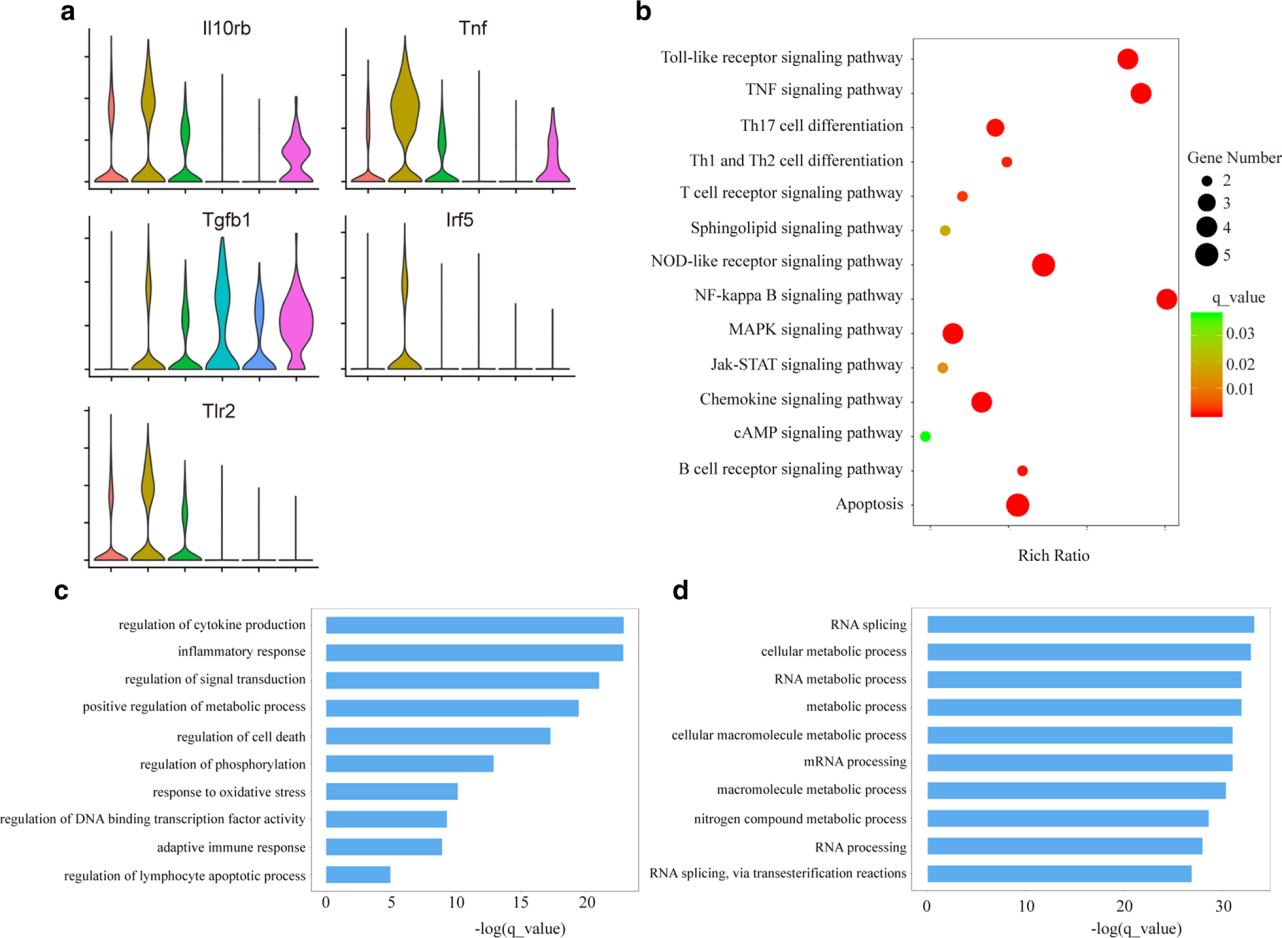
Changes on the biological function of PD-L1-expressed neutrophil subset. **a** Violin plot showing normalized expression of marker genes for the different subclusters. **b** KEGG pathway analysis for differently expressed mRNAs in cluster 2. **c**, **d** The enriched GO terms (biological processes) of differently expressed mRNAs in cluster 2 and cluster 4 are shown

We found that genes associated with immunoregulation (IL-10rb, Tnf, Tgfβ1, Irf5 and Tlr2) were expressed specifically in subcluster 2 (Fig. 3a). However, many genes with similar functions were not detected in the cells (data not shown). We identified the most significantly different genes in each subcluster (Additional file 1: Fig. S4A). To determine whether the PD-L1- and MHC-II-expressing subsets have immunoregulatory functions, GO analysis was performed. The results demonstrated highly significant overrepresentation of the regulation of the immune system in subcluster 2 (Fig. 3c), including the adaptive immune response, regulation of cytokine production, lymphocyte apoptotic process, cell death and phosphorylation. However, in subcluster 4, GO analysis demonstrated highly significant overrepresentation of the metabolic process (Fig. 3d). To identify signaling pathways involved in subcluster 2, we performed enrichment analysis of the KEGG pathways (Fig. 3b).

KEGG pathway analysis predicted activation of NF-kappa B-, TNF-, NOD-like receptor-, Toll-like receptor-, apoptosis-, MAPK- and Jak-STAT-mediated pathways. Thus, the PD-L1-expressing subset of neutrophils was indicated to have abnormal biological function in sepsis. This subtype was preliminarily considered as inhibitory neutrophils because of its immunomodulatory function.

### Sepsis results in neutrophil differentiation by the p38α-MSK1/-MK-2 pathway

**Fig. 4:**
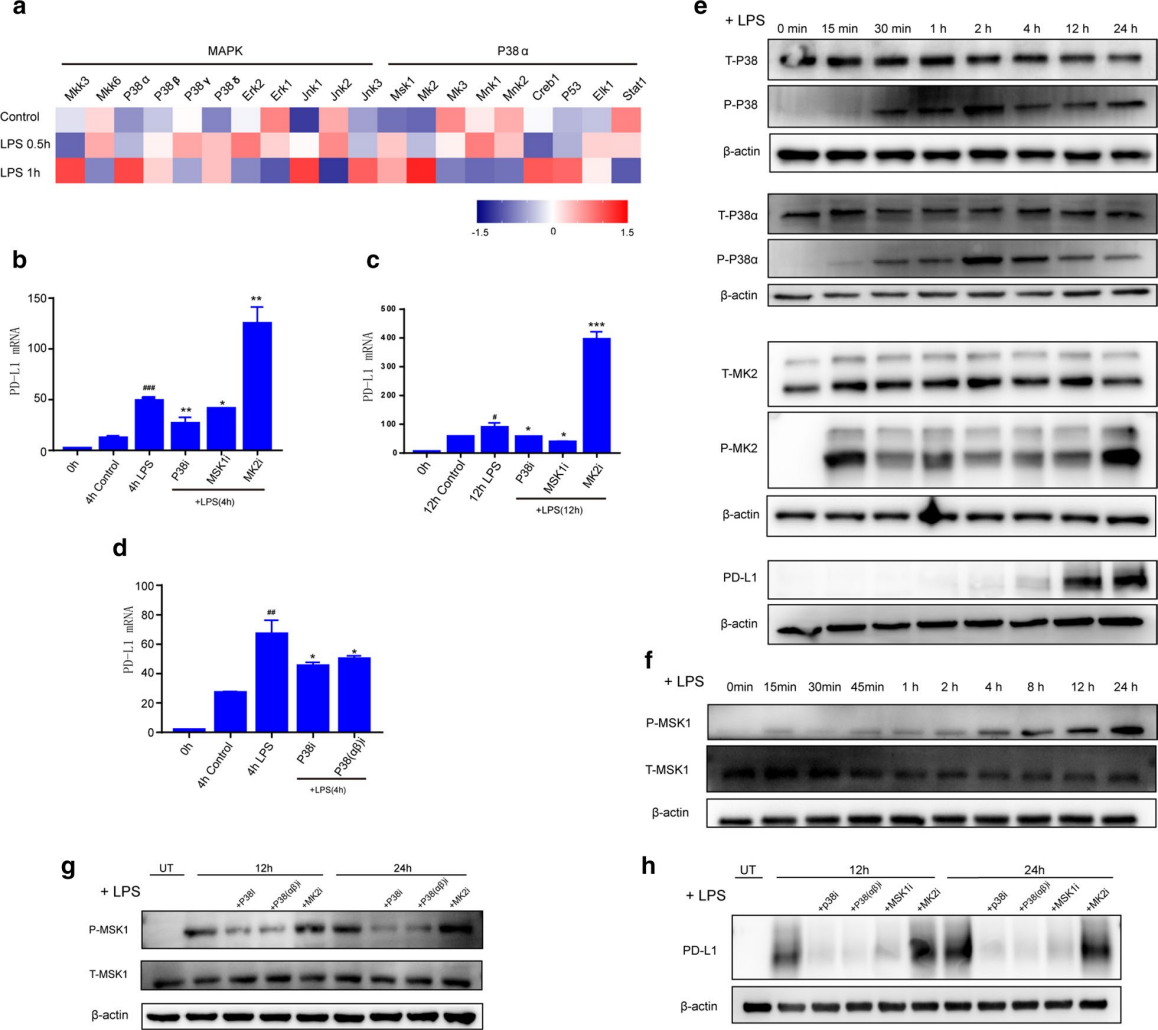
Sepsis induces neutrophil differentiation is mediated by p38α-MSK1/-MK2 pathway. **a** Gene chip showed activation of MAPK and p38α pathways at 0.5 h and 1 h after LPS (1 μg/mL) stimulation. **b**, **c** q-PCR results for PD-L1 mRNA expression. Neutrophils were challenged with LPS (1 μg/mL) or pre-treated with p38i (10 μM), MSK1i (25 μM) or MK2i (20 μM) prior to LPS challenge (n = 4). **d** q-PCR results for PD-L1 mRNA expression. Neutrophils were challenged with LPS (1 μg/mL) or pre-treated with p38i (10 µM), P38(αβ)i (10 µM) prior to LPS challenge (n = 4). **e**, **f** Different time points after LPS (1 μg/mL) stimulation of neutrophils, the phosphorylation of p38, p38α, MSK1, MK2 and expression of PD-L1 were observed. **g** Western blot results of the phosphorylation of MSK1 in neutrophils which stimulated with LPS (1 μg/mL) in 12 and 24 h. P38i (10 µM), P38(αβ)i (10 µM) or MK2i (20 µM) was preloaded. **h** Western blot results of PD-L1 expression in neutrophils which stimulated with LPS (1 μg/mL) in 12 h. p38i (10 µM), p38(αβ)i (10 µM), MSK1i (25 µM), MK2i (20 µM) were preloaded. **i** inhibitors. ^#^p < 0.05, ^##^p < 0.01 and ^###^p < 0.001, compared with control group; *p < 0.05, **p < 0.01 and ***p < 0.001, compared with LPS group. Data are mean ± SEM

To identify the cell signaling pathway that promotes neutrophil differentiation into a PD-L1-expressing subset, we challenged neutrophils with LPS for 15 min. We found that 4 h of LPS stimulation is long enough to induce PD-L1, which suggested that intracellular kinases might be involved in this process. As the KEGG analysis of cluster 2 showed that the MAPK pathway was significantly enriched (Fig. 3b), we used a gene chip to detect the transcription spectrum of neutrophils and demonstrated that the expression of p38α and its downstream factors MSK1 and MK2 were increased after LPS challenge for 0.5 and 1 h (Fig. 4a). Thus, we suggest that the p38α-MSK1/-MK2 pathway mediates neutrophil differentiation into a PD-L1-expressing subset.

To investigate the role of the p38α-MSK1/MK2 pathway, we introduced specific inhibitors for p38, MSK1 or MK2 to neutrophils and challenged them with LPS for 4 or 12 h. Subsequently, neutrophil PD-L1 mRNA expression was determined. As shown in Fig. 4b–d, inhibition of p38 (including p38αβ) and MSK-1 prevented LPS-induced PD-L1 mRNA expression in neutrophils. In contrast to p38 and MSK1, MK2 inhibition enhanced LPS-induced PD-L1 mRNA expression in neutrophils.

A time course study indicated that LPS increased the phosphorylation of p38 (including p38α), MSK1 and MK2 starting 15 min after LPS stimulation. Notably, MSK1 phosphorylation peaked at 12 to 24 h after LPS stimulation. LPS-induced PD-L1 protein expression started at 12 h and peaked at 24 h (Fig. 4e, f). Furthermore, neutrophils were pretreated with a specific inhibitor of p38, p38αβ or MK2, followed by LPS challenge for 12 h and 24 h, and neutrophil MSK1 phosphorylation was assessed. As shown in Fig. 4g, inhibition of p38 or p38αβ diminished LPS-induced phosphorylation of MSK1, while inhibition of MK2 enhanced LPS-induced phosphorylation of MSK1. Finally, inhibition of p38 or MSK1 attenuated the LPS-induced increase in PD-L1 expression in neutrophils, while inhibition of MK2 increased the LPS-induced PD-L1 expression in neutrophils (Fig. 4h).

### Inhibitory neutrophils induce T lymphocyte transdifferentiation and immunosuppression via the PD-L1 pathway

**Fig. 5:**
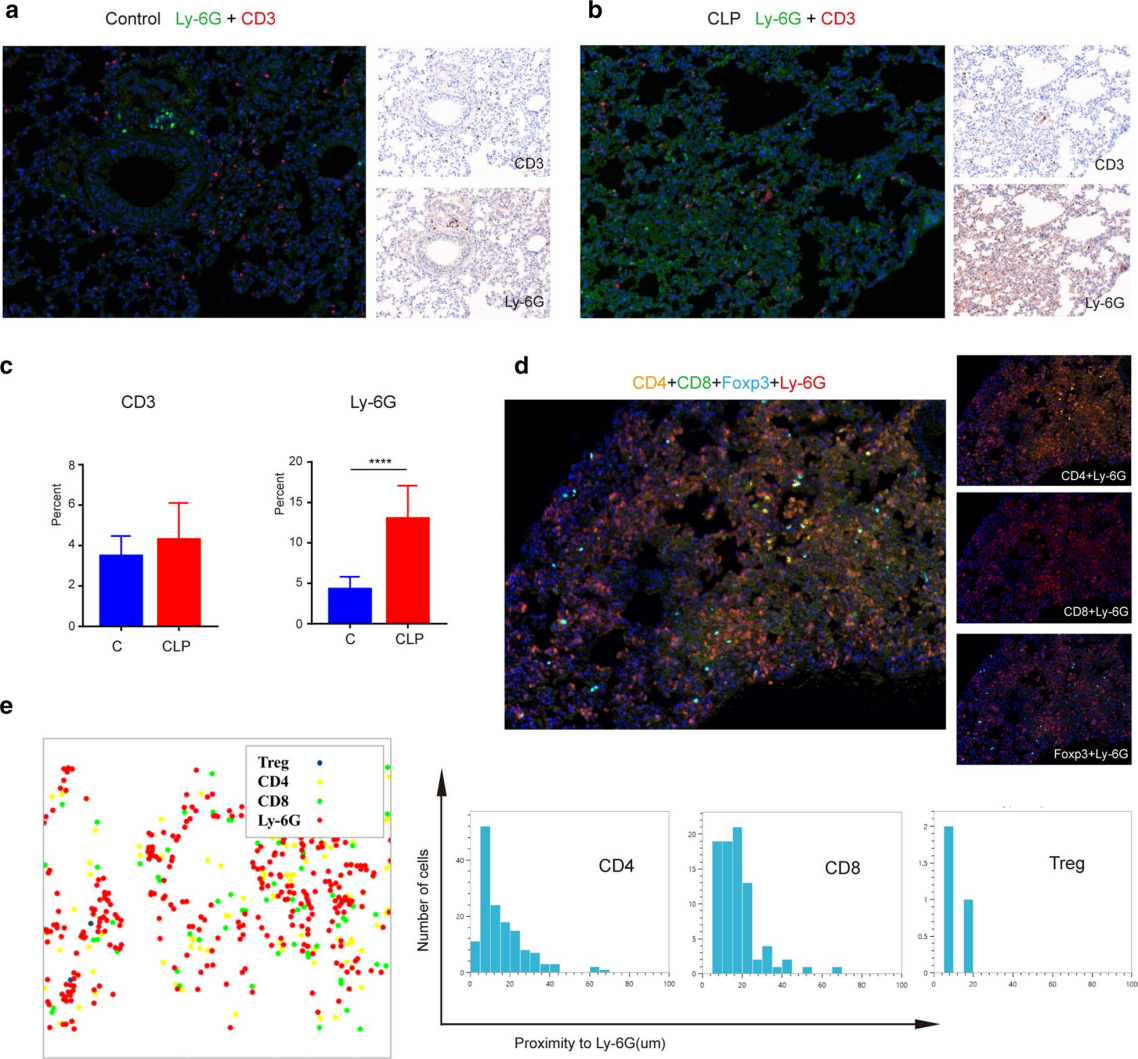
The tiny spatial distribution between neutrophils and regulatory T cells. **a**, **b** The immunofluorescence and immunohistochemical pictures of mouse lungs in control and CLP group. The neutrophils were stained in green by Ly-6G, and the T cell was stained in red by CD3. The results of single staining were presented on the right side. **c** Statistics of lymphocytes and neutrophils in control and CLP group (n = 12). **d** The immunofluorescence pictures of mouse lungs in CLP group. Red is Ly-6G labeled neutrophils, green is CD8 labeled CD8 lymphocytes, yellow is CD4 labeled CD4 lymphocytes, sky blue is Foxp3 labeled T-reg cells, blue is DAPI labeled nuclei. **e** Spatial distance between neutrophils and different lymphocytes. The cells were presented by dots with different colors. ****p < 0.0001. Data are mean ± SEM

To investigate the inhibitory function of neutrophils in sepsis, we performed neutrophil (Ly-6G) and T cell (CD3) staining to examine the immune status of the lungs of control and septic mice (Fig. 5a, b). As shown in Fig. 5c, the expression of Ly-6G was much higher in the lungs of CLP mice than in control mice (Fig. 5c). Interestingly, the patterns of neutrophils and lymphocytes were consistently distributed (Additional file 1: Fig. S5A-B). However, further study by labeling CD4+, CD8+ and Foxp3 + infiltrating cells in the lung showed that neutrophils and different subtypes of lymphocytes were differentially distributed (Fig. 5d). To determine the relationship between neutrophils and different lymphocyte subtypes, we analyzed the two-dimensional spatial distribution of neutrophils and lymphocytes. As shown in Fig. 5e and Additional file 1: Fig. S5C, neutrophils showed spatial isolation from CD4 + and CD8 + cells. Interestingly, the distribution of Foxp3 + cells (T-regs) was much closer to that of Ly-6G than CD4 + and CD8 + cells. These results indicate that neutrophils play an immunomodulatory role on surrounding T-regs.

**Fig. 6:**
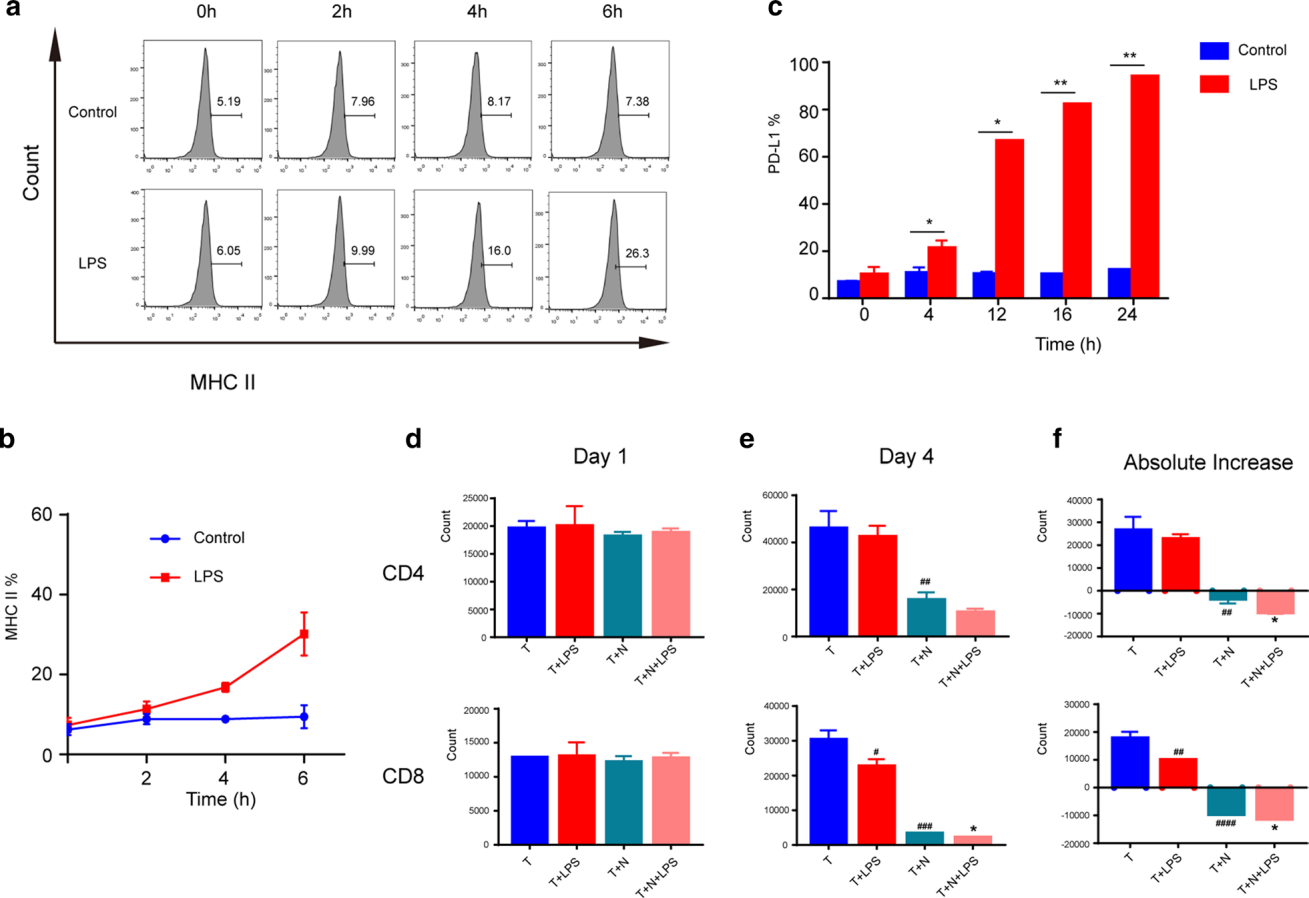
Effects of neutrophils on lymphocytes. **a**, **b** Changes in expression of MHC-II of neutrophils, which were stimulated with LPS (1 μg/mL) in 0, 2, 4 and 6 h. **c** Changes in expression of PD-L1 in neutrophils, which were stimulated with LPS (1 μg/mL) in 0, 4, 12, 16, 24 h (n = 3). *P < 0.05 and **P < 0.01, compared with control group (Student’s t test). **d**–**f** The effect of neutrophils on count of CD4 and CD8 cells. The neutrophils were stimulated with LPS (1 μg/mL) in advance for 4 h. CD4 and CD8 cells were detected on day one and day four of co-culture. The count change between day one and day four was also observed (n = 3). ^#^p < 0.05, ^##^p < 0.01, ^###^p < 0.001 and ^####^p < 0.0001 compared with *T* group; *p < 0.05, compared with *T* + *N* group

To investigate the potential modulatory role of neutrophils on T cells in sepsis, PD-L1 and MHC-II were analyzed with a time course study. As shown in Fig. 6a–c, the percentage of MHC-II and PD-L1 subset neutrophils gradually increased after LPS challenge. Furthermore, we explored T lymphocyte changes after coculture with neutrophils. T lymphocytes derived from mouse spleens were activated with CD3/CD28 and cocultured with LPS-challenged neutrophils for up to 4 days, and T lymphocyte counts were detected. As shown in Fig. 6d–f, the coculture of T lymphocytes with LPS-challenged neutrophils did not change on day 1. However, the CD4 + and CD8 + T cells were significantly decreased at day 4 of coculture.

**Fig. 7:**
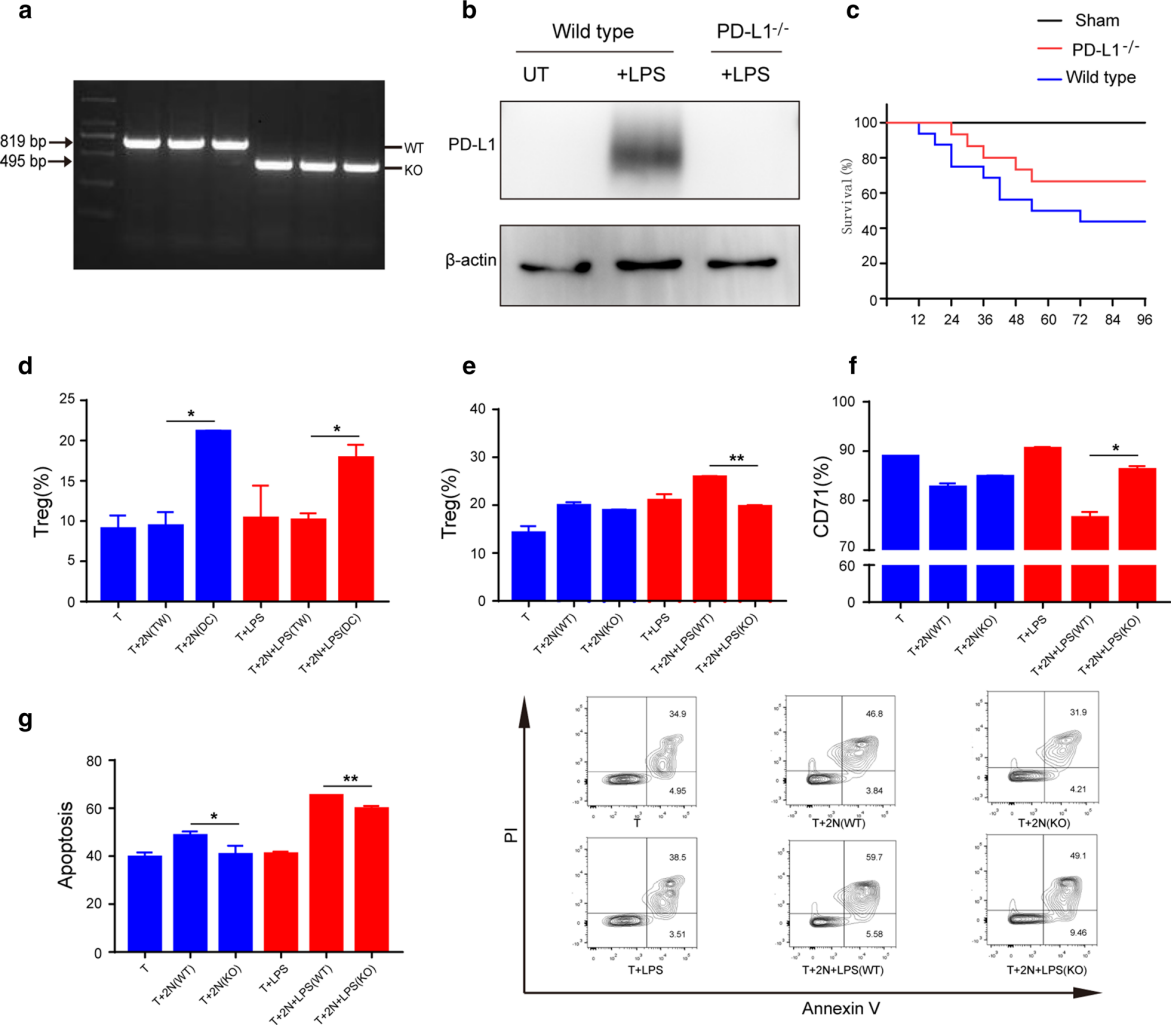
Inhibitory neutrophils induce T lymphocytes trans-differentiation and immunosuppression via PD-L1 pathway. **a** PD-L1 was knocked out (KO) in C57BL/6 mice. Expression of PD-L1 was detected by PCR. **b** LPS-stimulated wild type and PD-L1^−/−^ neutrophils for 12 h, and the expression of PD-L1 was detected by western blot. **c** Survival rate of wild type and PD-L1^−/−^ mice with sepsis. Sepsis was induced by CLP. Sixteen mice per group. **d** Effect of neutrophils on lymphocytes’ trans-differentiation. Lymphocytes co-culture with neutrophils in 4 days, neutrophils were stimulated with LPS (1 μg/mL) in 4 h. The results of transwell (TW) insert co-culture model were also observed (n = 4). **e** Effect of PD-L1-deficient LPS-stimulated neutrophils on lymphocytes’ trans-differentiation. Lymphocytes co-culture with neutrophils in 4 days, WT and PD-L1^−/−^ neutrophils were stimulated with LPS (1 μg/mL) in 4 h. The CD4, CD25 and FOXP3 were observed (n = 4). **f**–**g** Effect of PD-L1-deficient and LPS-stimulated neutrophils on lymphocytes’ activation and apoptosis (n = 5). Cell intervention and culture methods were consistent with **d**. *p < 0.05, **p < 0.01

Considering the importance of PD-L1 in neutrophil immunoregulation, we purchased PD-L1-deficient mice (on a C57BL/6 background, PD-L1^−/−^) (Fig. 7a, b). PD-L1^−/−^ mice were subjected to CLP. We found that the survival rate of PD-L1^−/−^ mice with sepsis was significantly improved (Fig. 7c). We cocultured T cells alone or together with neutrophils, and T-regs were detected. As shown in Fig. 7d, direct coculture of neutrophils with T cells resulted in an increased proportion of T-regs with the CD4^+^CD25^+^FOXP3^+^ phenotype. The effect was not observed when cells were cocultured with transwell inserts. This is direct evidence that neutrophils promote T lymphocyte differentiation through direct contact. However, when PD-L1^−/−^ neutrophils were cocultured with T lymphocytes (Fig. 7e), the neutrophil-mediated increase in T-regs was diminished. These results suggest that the modulatory function of neutrophils on lymphocyte transdifferentiation is dependent on neutrophil expression of PD-L1 and direct contact with the neutrophil.

In our direct coculture system, a large fraction of T lymphocytes were CD71^+^. However, coculture of LPS-treated neutrophils with lymphocytes greatly suppressed CD71 expression, but this effect was not observed in the presence of PD-L1-deficient neutrophils (Fig. 7f). Coculture of neutrophils with T cells significantly increased T cell apoptosis. The effect was diminished in the presence of the PD-L1-deficient neutrophils (Fig. 7g).

## Discussion

Neutrophils are professional killer cells in the innate immune system, mainly by phagocytosing, killing and digesting bacteria and fungi [10, 19]. Currently, increasing evidence verifies that neutrophils not only have direct antimicrobial ability but also participate in regulating the adaptive immune response [20–22]. The adaptability of neutrophils in the adaptive immune response has become a research hotspot. Although the heterogeneity of neutrophils in autoimmune diseases [23, 24] and the tumor microenvironment [25, 26] has been found, the basis for neutrophil classification has not been widely accepted. The classification of neutrophils in sepsis remains elusive. Here, we showed a inhibitory subset of neutrophils that was found by single-cell sequencing in both sepsis patients and mice. Inhibitory neutrophils highly express PD-L1 and suppress the innate immune response by upregulating the proportion of T-regs. Moreover, blocking PD-L1 expression on neutrophils effectively inhibits T-reg proliferation and reverses the immunosuppressive status in sepsis. Therefore, inhibitory neutrophils play an important role in the sepsis immunosuppressive response, and PD-L1 on neutrophils is a therapeutic target.

Neutrophils show bidirectional regulatory effects on the adaptive immune response [20, 21, 27]. They exert APC-like functions to upregulate immunoreactions and release cytokines, ROS and Arg-1 to inhibit T cell activation and proliferation. This evidence indicates that neutrophils possess different functional subgroups. In tumor immunity, tumor-associated neutrophils (TANs) can be divided into antitumor (N1) and permissive (N2) phenotypes [28, 29]. The N2 subset shows a protumorigenic function. In inflammatory infection, the classification of murine neutrophils has been reported in a mouse model of methicillin-resistant *Staphylococcus aureus* infection. That study found that neutrophils include three subtypes: PMN-N, PMN-I and PMN-II. PMN-II (the inhibitory subset) is involved in the generation of anti-inflammatory macrophages (M2) through IL-10- and CCL2-dependent mechanisms [30]. Previous studies have also shown that CD62L^low^CD11b^high^ subsets are highly correlated with inhibitory neutrophil phenotypes, such as CD244, CD115, CD11c, CD32, CD35, CD45 and CD66b [31]. Several studies have shown that specific subsets of neutrophils can inhibit lymphocyte function and play an immunosuppressive role [32, 33]. In human systemic inflammation, a specific phenotype of neutrophils (CD11c^bright^/CD62L^dim^/CD11b^bright^/CD16^bright^) can inhibit T cell proliferation through Mac-1 [32]. Jia-feng Wang et al. found that sepsis could induce increased expression of neutrophil PD-L1. By co-culture of neutrophils with lymphocytes, they found that neutrophils can induce apoptosis of lymphocytes through PD-L1-mediated direct contact mechanism. Therefore, PD-L1 is up-regulated on neutrophils during sepsis, which may be related to sepsis-induced immunosuppression [33]. Furthermore, granulocyte-derived suppressor cells (G-MDSCs) are considered to be precursor inhibitory neutrophils [5, 7]. However, whether this kind of low-density granulocyte (LDG) is a PMN is still not clear. Thus, there is still no sufficient evidence of an immunosuppressive neutrophil subset in sepsis, and this challenge urgently needs to be solved. With the discovery of regulatory B cells and regulatory NK cells that secrete IL-10 [34, 35], the existence of immunosuppressive neutrophils was suspected. However, human neutrophils cannot release IL-10, and this cytokine is only detected in a mouse model [36]. IL-10 is not a good marker to analyze immunosuppressive neutrophils.

In this study, we analyzed the biological behavior of neutrophils in sepsis and defined inhibitory neutrophils based on heterogeneity. First, the immunological plasticity of neutrophils in sepsis was studied, and single-cell gene sequencing technology was used to explore the immunological typing. LPS was used to stimulate neutrophils in vitro to simulate sepsis. After 4 h of stimulation, the cells were collected for complete transcriptome analysis. Based on single-cell gene sequencing analysis, neutrophils were divided into 6 subclusters. Each subcluster expressed characteristic genes, including PD-L1 and MHC-II, which are markers of immunosuppression and immune activation. Through GO analysis, the biological behavior of the high-expressing PD-L1 subcluster was analyzed, and it was found that the differential genes of this subcluster were significantly enriched in the process related to immune regulation, which provided sufficient evidence to explain the unique function of this subcluster. At the same time, PCR and western blotting results confirmed that the expression of PD-L1 was upregulated by activating the p38-MSK1/-MK2 signaling pathway in LPS-stimulated neutrophils in vitro. We hypothesized that neutrophils might suppress acquired immunity through the PD-L1/PD-1 immune checkpoint in sepsis.

Second, we investigated the effect of neutrophils on T cells during inflammation. T lymphocytes are an important part of the immune system, and previous experiments have shown that neutrophils inhibit T cells by consuming essential amino acids (such as l-arginine) from the microenvironment, producing ROS or intercellular contact. Surprisingly, our study found that neutrophils induced T-reg production by direct contact with spleen-derived lymphocytes, which was not observed in transwell models. This showed the importance of direct contact between neutrophils and lymphocytes. Moreover, we collected neutrophils from wild-type and PD-L1 knockout mice and cocultured them with lymphocytes from wild-type mice with or without LPS treatment. We found that the number of T-regs in the knockout group was significantly lower than that in the wild-type group (19.6% vs. 25.8%). We also examined apoptosis and the activation marker CD71 of T lymphocytes after coculture and found that wild-type mouse neutrophils stimulated by LPS-induced T cell apoptosis and inhibited activation. Immunosuppression was partially attenuated after PD-L1 knockout. Therefore, there is good reason to think that neutrophils play an immunosuppressive role in infection by influencing a variety of biological behaviors of T cells, in which PD-L1 plays a crucial role.

Interestingly, when we measured the spatial distance between the lung neutrophils and different subtypes of lymphocytes in CLP mouse lungs, we found for the first time that FOXP3^+^ lymphocytes were more closely distributed to neutrophils than other T lymphocytes. This appears to be morphological evidence that neutrophils induce T-reg formation by direct contact.

**Fig. 8:**
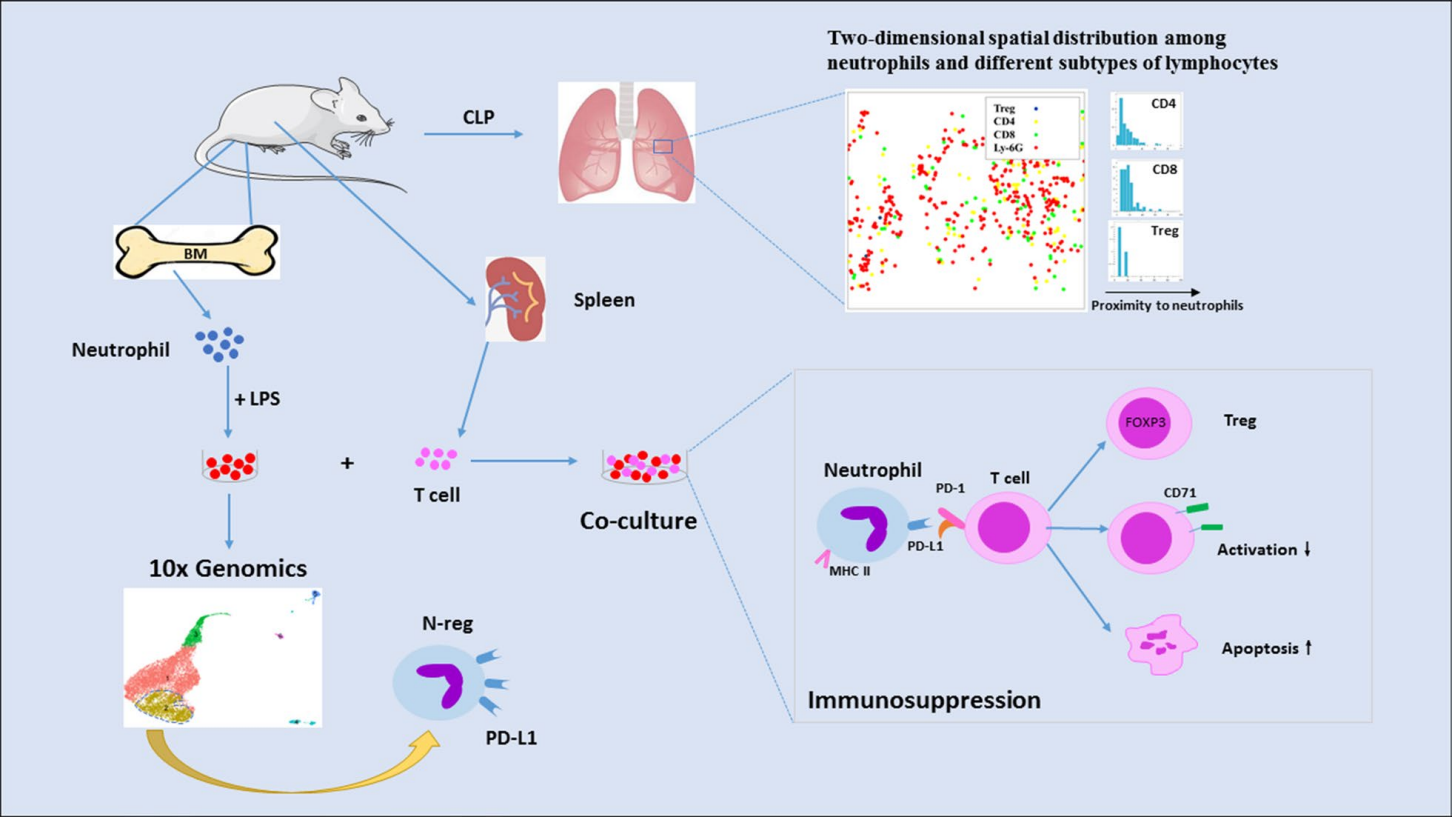
Schematic illustration of the mechanism of inhibitory neutrophils mediating immunosuppression. Single-cell RNA sequencing confirmed that neutrophils were divided into multiple subclusters in sepsis. LPS mediates PD-L1 expression through p38α-MSK1/-MK2 pathway in neutrophils, and the subsets of highly expressed PD-L1 exert immunosuppressive function, including inducing T cell trans-differentiation, promoting T cell apoptosis and inhibiting T cell activation

Our definition of inhibitory neutrophils based on neutrophil heterogeneity is more focused on the plasticity of immune cells than the existing definition of neutrophil subsets. In other words, neutrophils are homogeneous with inflammatory phenotypes before activation, and heterogeneous neutrophils are found in sepsis, which further emphasizes the use of the functional status of cells as the classification standard of cell subgroups (Fig. 8). Our previous studies have shown that neutrophil chemotaxis is inhibited by LPS, which may be one of the important characteristics of inhibitory neutrophils [12]. Our study is also the first to overcome the difficulty of rapid neutrophil apoptosis and in-depth evaluation of neutrophil heterogeneity in the sepsis microenvironment at the level of the single-cell transcriptome.

Single-cell genomics has opened up a new view of immunity [37–39]. Traditional methods cannot parse the complex immune landscape. Almost all of the textbook definitions and stereotypes of cellular identity will need to be reconsidered. Our experimental study was based on the deficiencies in neutrophil immunotyping and used single-cell RNA sequencing technology to analyze purified neutrophils. Guided by biological behavior, we analyzed the neutrophil subsets that negatively regulate acquired immunity. However, our study also has limitations. On the one hand, in in vivo experiments, adolescent male mice were selected for CLP. An international expert consensus recommends that the design of the sepsis model should consider replication of the findings in models that include some biological variables (i.e., age, gender and others) [40]. Therefore, different ages and both sexes should be considered in future experiments. The experts also recommend that microorganisms used in models preferentially replicate those commonly found in human sepsis and consider initiating sepsis syndrome modeling in areas other than the peritoneal cavity (e.g., lung, urinary tract, brain). At the same time, fluid resuscitation and antimicrobial were also recommended, which makes the pre-clinical study more close to the clinical practice [40]. On the other hand, we used LPS to induce a sepsis in vitro, but the subtype of neutrophil in response to multiple cell infection states remains unknown. LPS stimulation in vitro is only one of the current research methods, because this model is quite different from other animal models of sepsis, especially from sepsis patients. Furthermore, although inhibitory neutrophils have been shown to induce immunosuppression in vitro, there is no evidence in vivo.

## Conclusions

In general, the discovery and naming of inhibitory neutrophils will facilitate the study of specific neutrophils, including how to prevent neutrophils from entering an immunosuppressive state early in infection and how to eliminate immunosuppressive neutrophils late in inflammation. These further studies will lay the foundation for better understanding of the role of neutrophils in innate immunity and provide new ideas and intervention methods for the treatment of severe infection and sepsis. In fact, like other immune cells, the evolution of the biological behavior of neutrophils during severe infection is quite complex and requires further study in more dimensions.

## Supplementary information

**Additional file 1**. Supplemental data (Fig. S1-S6).

## Data Availability

The datasets used and/or analyzed in the current study are available by the corresponding author upon reasonable request.
