# Supplementary material for: Identification and characterization of neutrophil heterogeneity in sepsis
Source: Crit Care. 2021 Feb 6;25:50. doi: 10.1186/s13054-021-03481-0 (PMC7865119; doi:10.1186/s13054-021-03481-0)
Supplement: Supplementary file 1 — Additional file 1. Supplemental data (Fig. S1-S6). [file 13054_2021_3481_MOESM1_ESM.pdf]

Additional files

Title：Identification and characterization of neutrophil heterogeneity in sepsis

Authors: Xinxin Qi, Yao Yu, Ran Sun, Jiamin Huang, Lu Liu, Yunxi Yang, Tao Rui, Bingwei Sun

Correspondence to: Professor Bingwei Sun, MD, PhD, Department of Burns and Plastic Surgery, Affiliated Suzhou Hospital of Nanjing Medical University, Suzhou 215002, Jiangsu Province, China

Telephone: +86 512 6236 3018 **Fax:** +86 512 6236 3018

Email: sunbinwe@hotmail.com

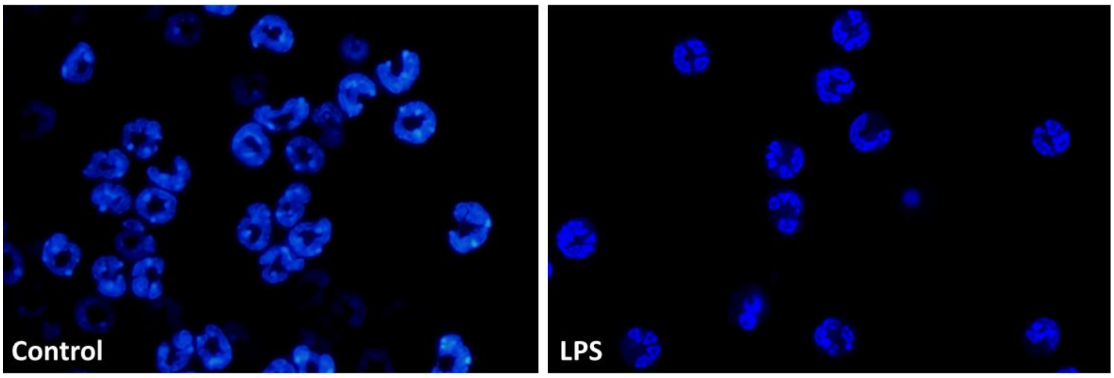

Additional files Fig. 1 Karyotype change of LPS-treated neutrophils were observed by Laser scanning confocal microscopy (LSCM). Neutrophils from mouse bone marrow were incubated in the presence and absence of LPS. LSCM showed changes in the neutrophil karyotype after 12 hours.

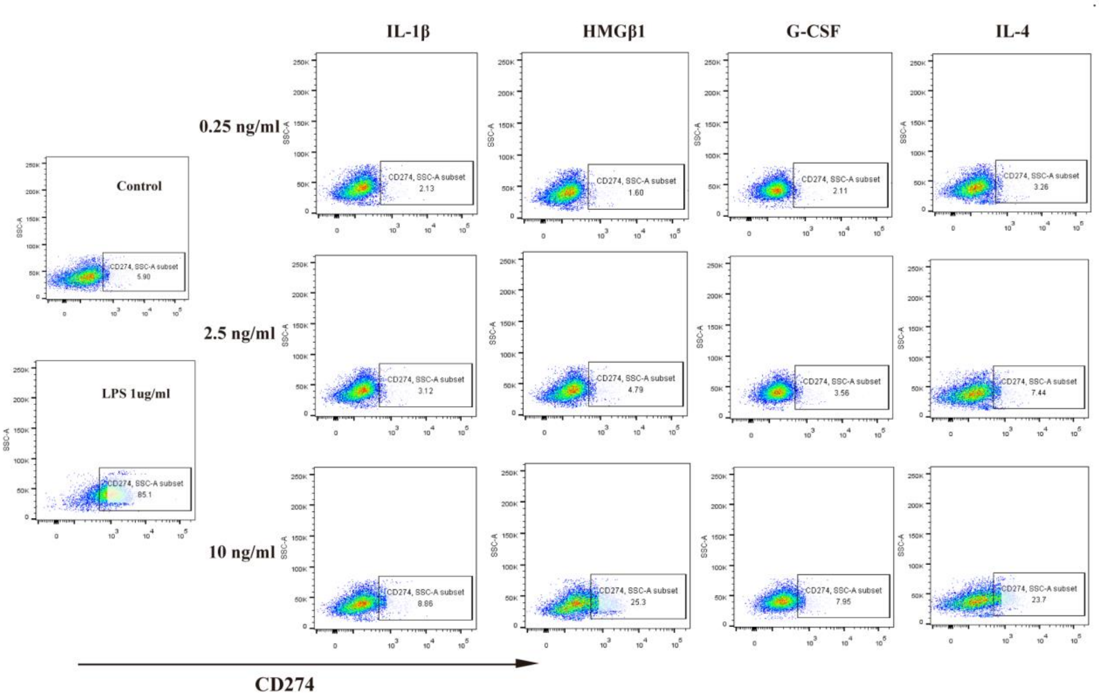

Additional files Fig. 2 Expression of PD-L1 in different co-culture conditions. PD-L1 expression of neutrophil under different stimuli were detected by flow cytometry. LPS (1ug/ml), IL-1β (0.25, 2.5, 10ng/ml), HMGβ1(0.25, 2.5, 10ng/ml), G-CSF (0.25, 2.5, 10ng/ml), and IL-4 (0.15, 1.5, 10ng/ml) stimulated the neutrophils.

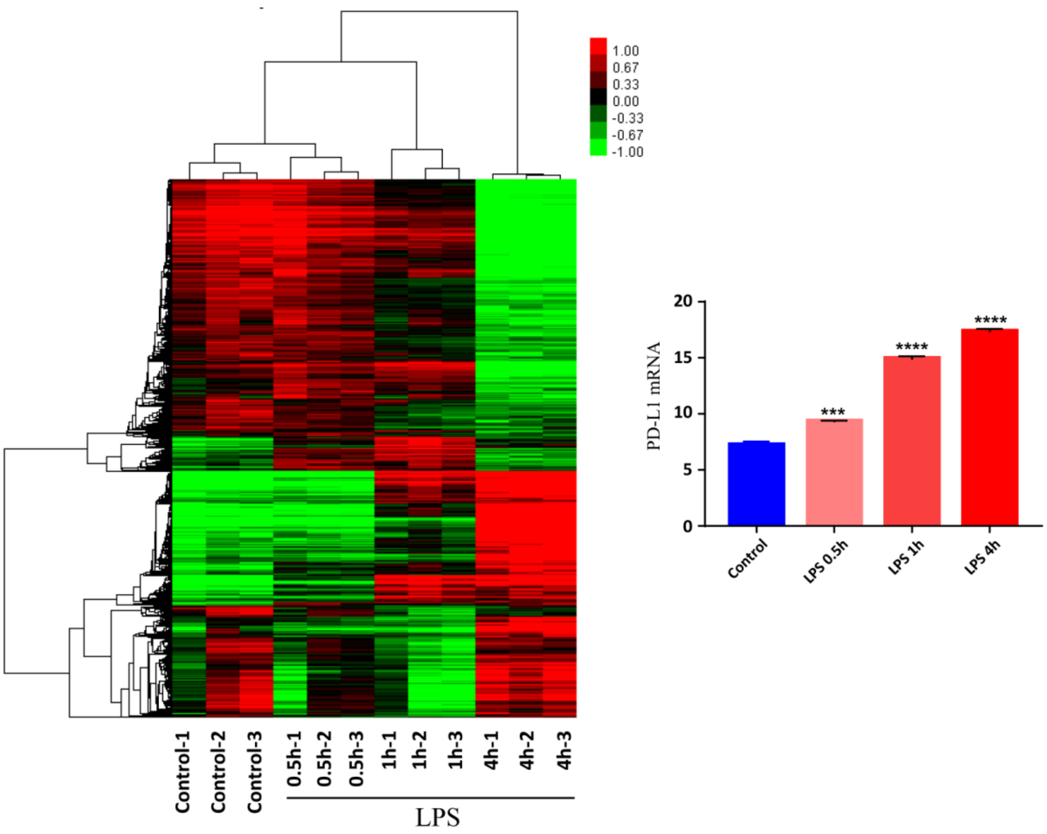

Additional files Fig. 3 Gene chip analysis of neutrophils after LPS intervention. Heat map shows the most differentially variable genes in control and LPS(0.5h, 1h, 4h) groups (left). Bar plot showing PD-L1 expression in neutrophils (right). ***p < 0.001 and ****p < 0.0001 compared with control group, Data are mean ± SEM (Student's t test).

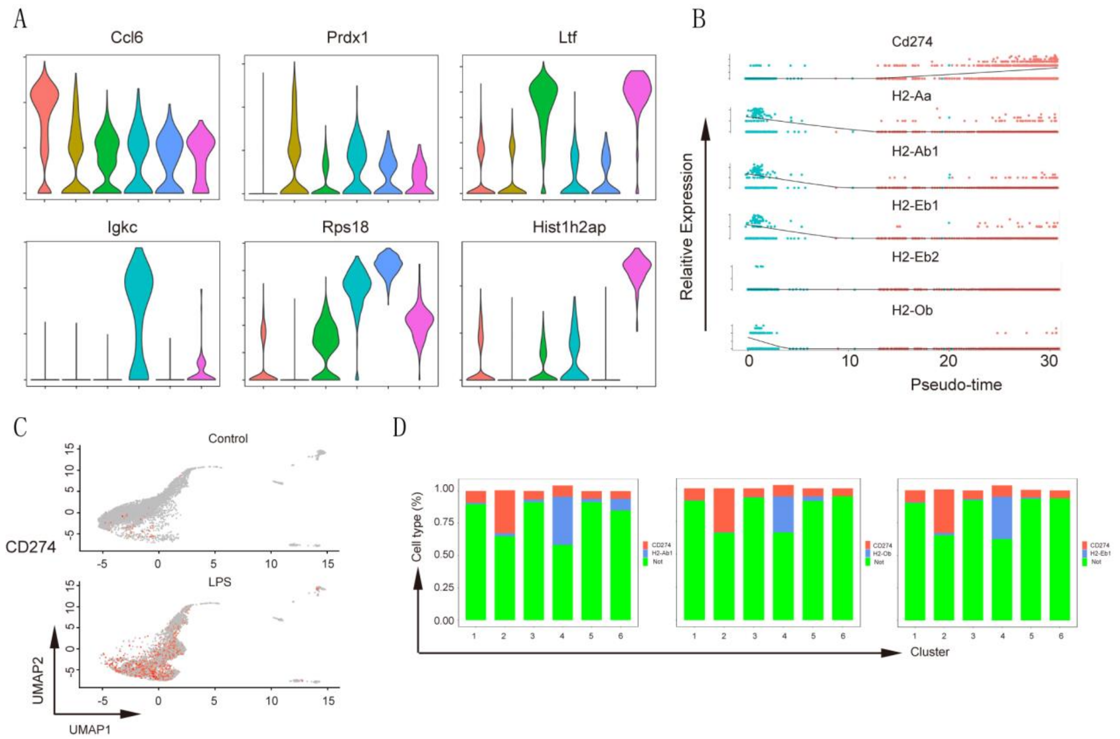

Additional files Fig. 4 The expression of marker genes for the different sub-clusters. (A) Violin plot showing normalized expression of marker genes for the different sub-clusters. (B) Representation of PD-L1 gene and MHC-Ⅱ components expression plotted as a function of pseudotime. (C) UMAP plots showing expression of PD-L1 in the subclusters. (D) Bar plot showing the proportions of neutrophils expressing PD-L1 and MHC-Ⅱ components (H2-Ab1, H2-Ob, H2-Eb1) in each clusters.

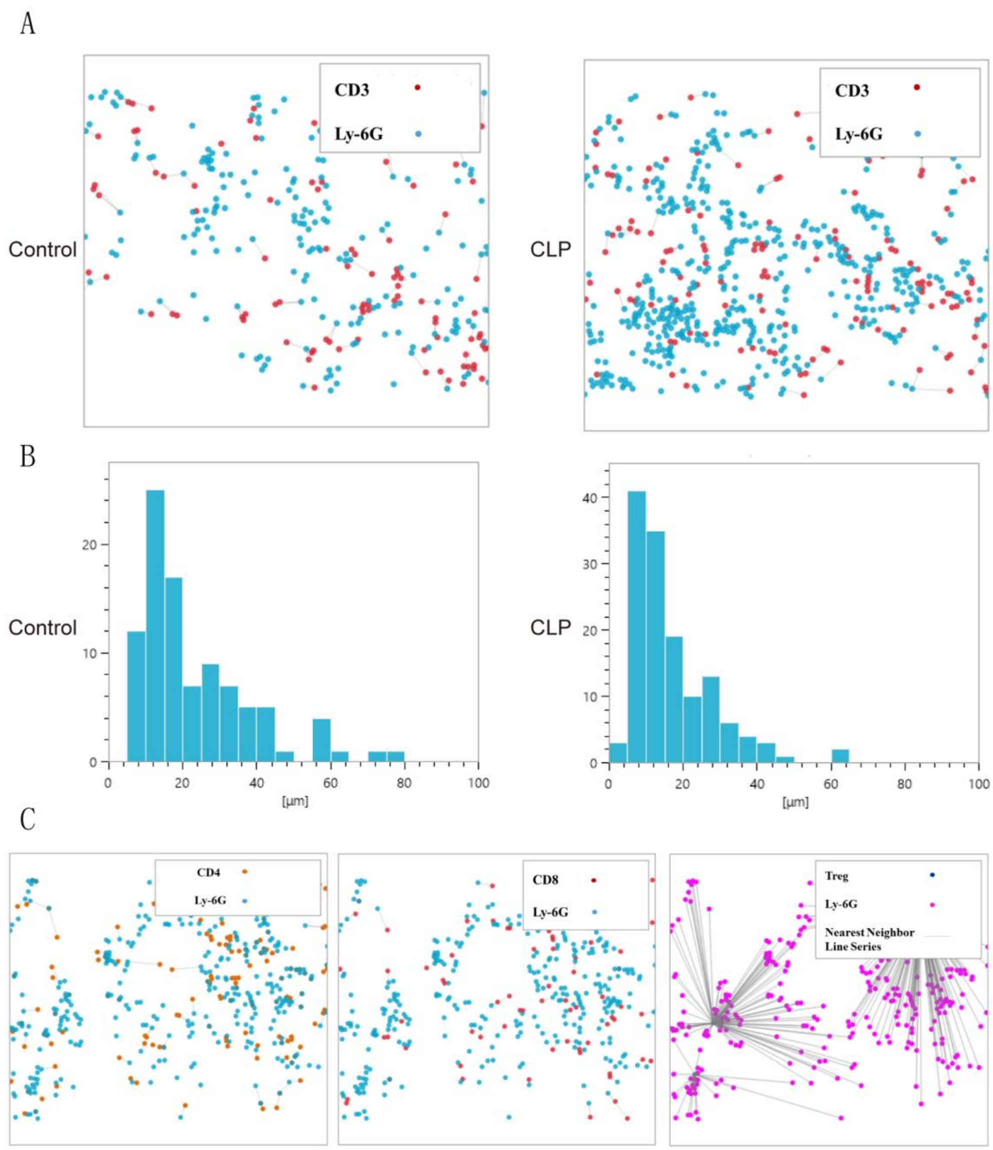

Additional files Fig. 5 Two-dimensional spatial distribution among neutrophils and lymphocytes. (A-B) Spatial distance between neutrophils and T lymphocytes. The cells were presented by dots with different colours. (C) The definition of nearest neighbor relationships between neutrophils and different lymphocytes. The cells were presented by dots with different colours.

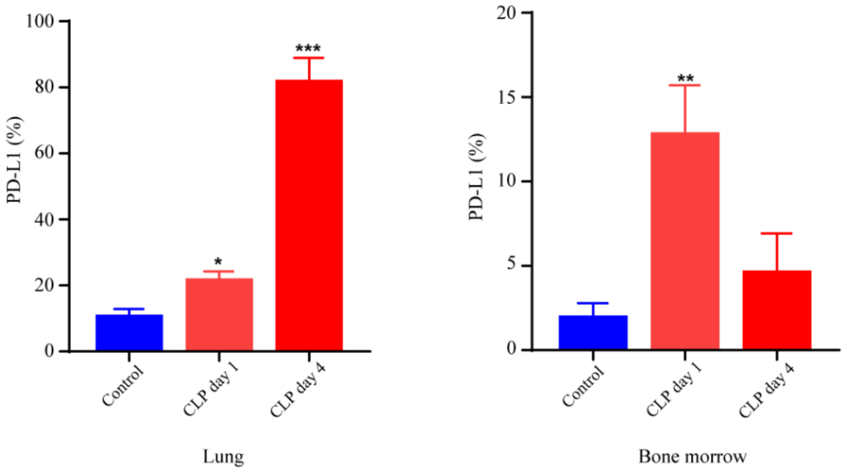

Additional files Fig. 6 PD-L1 expression in lung and bone morrow at different sepsis stages. One day and four days after CLP, the expression of PD-L1 on the surface of neutrophils was detected by flow cytometry. *p < 0.05 and **p < 0. 01 compared with control group，Data are mean ± SEM (Student's t test).
